# Secondary Intrascleral Intraocular Lens Fixation With Lens Capsule Preservation for Aphakic Eyes in Patients With Pseudoexfoliation Syndrome: A Case Series

**DOI:** 10.7759/cureus.70688

**Published:** 2024-10-02

**Authors:** Suguru Nakagawa, Satoru Kanda, Kiyoshi Ishii

**Affiliations:** 1 Ophthalmology, Saitama Red Cross Hospital, Saitama, JPN

**Keywords:** case series, iris fluttering, secondary surgery, intrascleral intraocular lens fixation, lens capsule preservation, zonule weakness, pseudoexfoliation syndrome, aphakic eyes, cataract surgery

## Abstract

We describe three cases of pseudoexfoliation syndrome (PEX) in which good outcomes were achieved after secondary intrascleral intraocular lens (IOL) fixation with capsule preservation for aphakic eyes. Three Japanese patients with PEX underwent phacoemulsification and aspiration (PEA) owing to challenges in IOL intracapsular fixation caused by zonular weakness. Case 1 involved an 83-year-old man with PEX. Six weeks post-PEA, 30-gauge needles were inserted to exit between the capsule and the iris. The IOL was fixed intrasclerally using the double-needle technique. Case 2 involved a 90-year-old man with PEX. The same abovementioned double-needle intrascleral IOL fixation procedure was performed eight weeks post-PEA. Intraoperative vitreous prolapse into the anterior chamber was observed, and anterior vitrectomy was performed. Case 3 involved an 80-year-old man with PEX. Seven weeks post-PEA, the patient underwent the same double-needle intrascleral IOL fixation procedure. Good IOL fixation was achieved in all patients without postoperative iris capture. No serious complications, including retinal detachment and vitreous hemorrhage, were observed. Preserving the capsule during secondary IOL scleral fixation for aphakic eyes can effectively reduce intraoperative vitreous prolapse, minimize surgical invasiveness, suppress iris flutter, and prevent capture of the pupillary IOL, making it a meaningful and acceptable approach, although the long-term risks, such as potential lens capsule drop, should be studied further.

## Introduction

Phacoemulsification combined with intraocular lens (IOL) implantation in the lens capsule represents the standard approach for cataract surgery. However, achieving this goal can be challenging in eyes with zonulopathy, such as zonular weakness and zonular dehiscence, where adequate zonular support for the capsule is lacking. During cataract surgery, upon encountering challenges such as zonular weakness, zonular dehiscence, and posterior capsular rupture, intracapsular fixation of the IOL can become difficult. As a result, the surgery may conclude with the removal of the cataract alone, resulting in an "aphakia," not having a lens inside the operated eye. When the surgery is performed on aphakic eyes, a second surgery becomes necessary for both sutured [1] or sutureless [2,3] intrascleral fixation of the IOL [4] in the absence of capsular support. In such cases, achieving good fixation of the IOL and preventing serious postoperative complications, such as retinal detachment, typically involves the removal of the remaining capsule followed by vitrectomy. The need for both resection of the capsule and vitrectomy increases the duration and complexity of the surgery, particularly for cataract surgeons.

Capsule resection can cause excessive flattening of the iris, potentially resulting in pupillary IOL capture or reverse pupillary block, which represents a major complication after IOL scleral fixation [5]. Reverse pupillary block may increase intraocular pressure (IOP) and induce uveitis-glaucoma-hyphema syndrome [5]. Although there are studies on IOL intrascleral fixation with capsule-sparing and IOL optic capture through anterior continuous circular capsulorhexis (CCC) in one-stage cataract surgery [6,7], to the best of our knowledge, there are no reports on IOL intrascleral fixation in secondary surgery with capsule preservation for aphakic eyes in which the initial cataract surgery was completed in the aphakic eye.

Herein, we report three cases of pseudoexfoliation syndrome (PEX) where the initial cataract surgery was completed on aphakic eyes with phacoemulsification and aspiration (PEA) due to difficulties in IOL insertion attributable to zonule weakness. Subsequently, during secondary IOL intrascleral fixation for the aphakic eye, intrascleral IOL fixation with capsule preservation was successfully performed, resulting in favorable outcomes among all individuals.

## Case presentation

All three patients had PEX and poor mydriasis, with a suspicion of zonular weakness. None of the cases exhibited significant systemic conditions such as diabetes or hypertension.

Case 1

An 83-year-old man was referred to our department for cataract surgery. His best-corrected visual acuity (BCVA) was 20/25 in the right eye and 20/20 in the left eye prior to surgery. IOP measured 19 mmHg in the right eye, controlled with 0.02% omidenepag isopropyl eyedrops, and 21 mmHg in the left eye. PEX was observed in the right eye, which also presented with nuclear cataracts of grade 2 and cortical cataracts. The right eye exhibited poor mydriasis, with a pupil diameter of approximately 3-4 mm. No retinal or vitreous disease was detected. Glaucomatous changes were noted in the right eye, characterized by thinning of the retinal nerve fiber layer (RNFL) at the 11 o'clock and 6-7 o'clock positions. The corneal endothelial cell density in the right eye was 2,678 cells/mm^2^.

**Figure 1:**
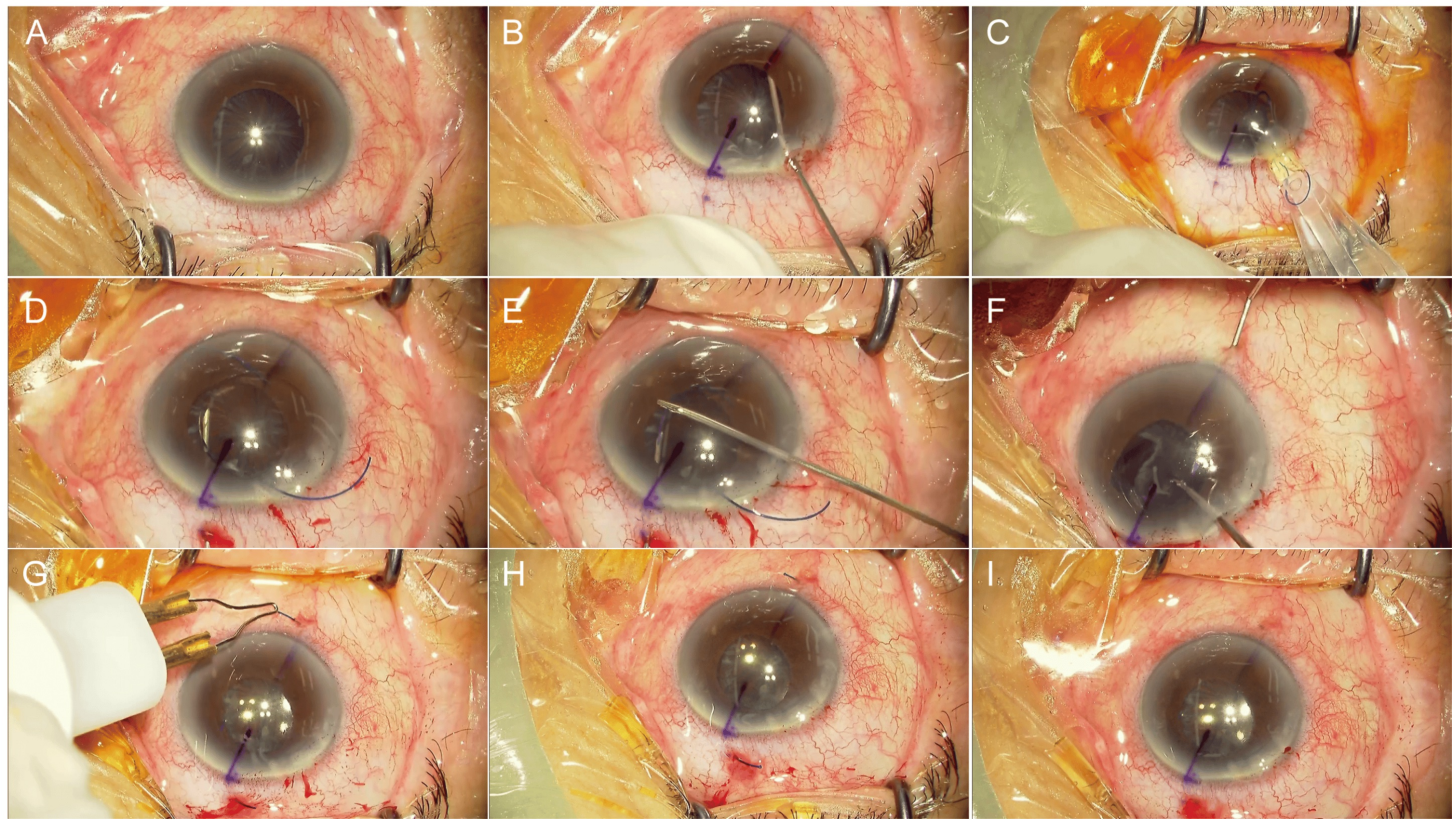
Intraoperative observations during intrascleral intraocular lens (IOL) fixation surgery in Case 1. IOL intrascleral fixation was performed six weeks after the initial cataract surgery (phacoemulsification). (A) Findings at the beginning of surgery. The lens capsule remained in place with shrinkage and opacity. (B) Adequate space was created between the capsule and the iris using a viscoelastic material. (C) A three-piece IOL was inserted with an injector; then, a 30-G needle was inserted 2 mm from the limbus such that the needle would exit between the capsule and the iris, and the IOL was fixed intrasclerally with the double-needle technique (D–H). (I) As no vitreous prolapse was observed intraoperatively, vitrectomy was not performed, and the capsule was left intact.

Due to inadequate pupil dilation, the initial surgery was performed using a Malyugin pupil dilator ring. He underwent surgery for a cataract of the right eye. The nucleus and cortex of the lens were removed; however, because of extensive dehiscence of the zonule from the 4 o'clock position to the 11 o'clock position, IOL insertion was expected to be difficult, even with the insertion of a capsular tension ring (CTR). Thus, surgery was terminated without IOL implantation. No intraoperative vitreous prolapse was observed, and the lens capsule was left intact. The postoperative BCVA was 0.9 in the right eye two weeks after the initial surgery. IOP was 16 mmHg in the right eye nine days postoperatively, controlled with 1% brinzolamide, 0.5% timolol maleate, and 0.1% brimonidine eye drops. Six weeks later, intrascleral IOL fixation was performed (Figure 1) (Video 1). A 30-gauge (G) needle was inserted 2 mm from the limbus such that the needle would exit between the lens capsule and the iris, and the IOL was fixed intrasclerally using the double-needle technique (Figures 1D-H). As no vitreous prolapse was observed intraoperatively, vitrectomy was not performed, and the lens capsule was left intact.

**Video 1:** Intrascleral intraocular lens fixation with lens capsule preservation for aphakic eyes in Case 1.

**Figure 2:**
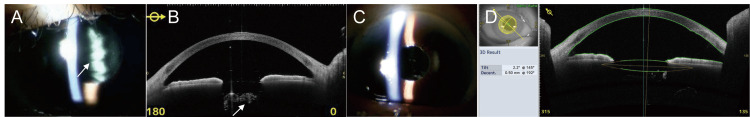
Postoperative findings after intrascleral intraocular lens (IOL) fixation surgery in Case 1. Anterior segment photograph (A) and anterior segment optical coherence tomography (OCT) image (B) obtained three months after the surgery. Capsular opacity over the visual axis (A, B, arrow) can be observed. Anterior segment photograph (C) and anterior segment OCT image (D) obtained at nine months postoperatively. No capsular opacity over the visual axis was observed. The anterior segment OCT image shows an IOL tilt (Tilt) of 2.2° and decentralization (Decent.) of 0.50 mm without iris capture by the IOL (D).

Eight days after the second surgery, the corneal endothelial cell density was 2,616 cells/mm^2^, the BCVA was 20/60, and the IOP was 16 mmHg. One month postoperatively, the BCVA improved to 20/30, and the IOP remained stable at 16 mmHg. Three months after the second surgery, the BCVA in the operated eye was 20/25. Capsular opacity was observed over the visual axis (Figures 2A, 2B). At both 18 weeks and six months postoperatively, an yttrium aluminum garnet (YAG) laser incision was made over the visual axis to treat the opacity of the capsule. Postoperatively, good IOL fixation was achieved without iris capture related to the IOL (Figure 2C). At nine months postoperatively, anterior segment optical coherence tomography (OCT) revealed an IOL tilt of 2.2° and decentralization of 0.50 mm (Figure 2D). Good visual acuity was achieved after the YAG laser capsulotomy. The BCVA at nine months postoperatively was excellent (20/16). Eight months after the surgery, the corneal endothelial cell density was 2,464 cells/mm^2^, representing a 6.5% overall decrease compared to the preoperative value.

Case 2

A 90-year-old Japanese man was referred to our department for cataract surgery in the right eye. The preoperative corneal endothelial cell density of the right eye was 2,538 cells/mm^2^. BCVA was 20/100, IOP was 17 mmHg, and pupil diameter was 5.6 mm. The patient had a grade 2.5 nuclear cataract, and PEX was observed in the right eye. No retinal or vitreous disease was present, and there were no glaucoma changes in the optic disc. The initial cataract surgery in the right eye was performed using a Malyugin pupil dilator ring to achieve adequate pupil dilation. A capsular stabilization device was used to remove the nucleus and cortex of the lens. However, owing to the potential presence of full-circumferential zonular weakness or dehiscence, IOL insertion was deemed challenging, even with the insertion of a CTR. Consequently, the surgery was completed without implanting the IOL. The lens capsule was left in place. No vitreous prolapse was noted.

**Figure 3:**
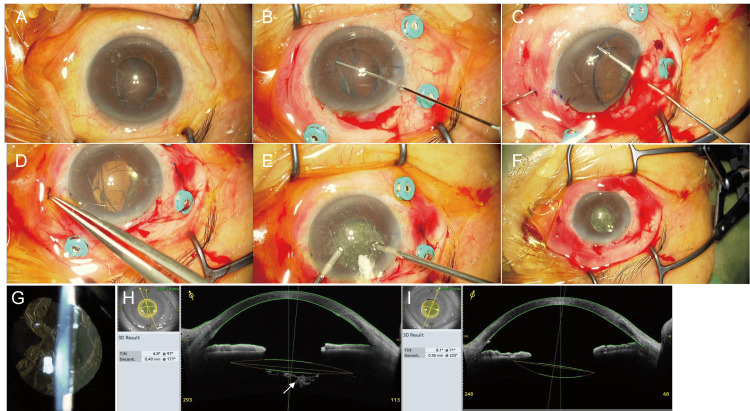
Intraoperative observations during intrascleral intraocular lens (IOL) fixation surgery in Case 2 and postoperative findings after IOL fixation surgery in Case 2. (A–F) IOL intrascleral fixation was performed eight weeks after the initial cataract surgery (phacoemulsification). (A) Findings at the beginning of surgery. The capsule remained in place, with some shrinkage and opacity. (B) Adequate space was created between the capsule and the iris using a viscoelastic material. (C,D) After inserting a three-piece IOL with an injector, a 30-G needle was inserted 2 mm from the limbus such that the needle would exit between the lens capsule and the iris, and the IOL was fixed intrasclerally using the double-needle technique. (E) During the surgery, vitreous prolapse into the anterior chamber was observed, possibly due to the IOL touching the capsule. Anterior vitrectomy was performed to remove the prolapsed vitreous from the anterior chamber. (F) The capsule was preserved. (G–I) Postoperative findings after IOL fixation surgery in Case 2. Anterior segment photograph (G) and anterior segment optical coherence tomography (OCT) image (H) obtained at six weeks postoperatively. Capsular opacity can be observed over the visual axis (H, arrow). Anterior segment OCT image (I) obtained at six months postoperatively. No capsular opacity was observed over the visual axis. The anterior segment OCT image shows an IOL tilt (Tilt) of 8.1° and decentralization (Decent.) of 0.56 mm without iris capture by the IOL (I).

One week after the initial surgery, the BCVA was 20/20, the IOP was 16 mmHg, and the corneal endothelial cell density was 2,223 cells/mm^2^. Eight weeks later, intrascleral fixation of the IOL was performed (Figure 3) (Video 2). As in Case 1, a 30-G needle was inserted such that it would exit between the capsule and the iris, and the IOL was fixed in the sclera using the double-needle technique (Figures 3B-D). At that time, vitreous prolapse into the anterior chamber was observed. Consequently, an anterior vitrectomy was performed (Figure 3E), and the lens capsule remained intact (Figure 3F). Five days after the second surgery, the BCVA was 20/60, IOP was 13 mmHg, and the corneal endothelial cell density could not be measured. YAG laser incisions were made over the visual axis to address the opacity of the capsule at both five days and six weeks postoperatively (Figures 3G, 3H). The BCVA of the operated eye prior to YAG laser incisions at six weeks was 20/30. Postoperatively, good IOL fixation was achieved without inadvertent iris capture. Six months after the surgery, anterior segment OCT showed an IOL tilt of 8.1° and decentralization of 0.56 mm (Figure 3I). Good visual acuity was achieved after YAG laser irradiation. The BCVA of the operated eye at six months postoperatively was 20/20. Six months after the surgery, the corneal endothelial cell density was 1159 cells/mm^2^, representing an overall decrease of 47.9% compared to the preoperative value.

**Video 2:** Intrascleral intraocular lens fixation with lens capsule preservation for aphakic eyes in Case 2.

Case 3

An 80-year-old Japanese man underwent surgery for a cataract in his right eye at another hospital. Zonular dehiscence was observed, and it was difficult to insert an IOL even with the use of a CTR. Consequently, a CTR was not implanted, and the surgery was terminated without IOL implantation. No intraoperative vitreous prolapse was observed, and the lens capsule was left intact. The patient's preoperative BCVA, IOP, and the degree of cataract were not documented as the initial surgery was performed elsewhere. The patient was referred to our department after the initial surgery, presenting with a BCVA of 20/100, an IOP of 11 mmHg with 0.5% timolol maleate eye drops, and a pupil diameter of 3 mm. The right eye was aphakic with an intact lens capsule and no vitreous prolapse in the anterior chamber. PEX was observed in both eyes, and the corneal endothelial cell density was 1,969 cells/mm^2^. There were no retinal or vitreous diseases, but glaucomatous changes were noted, with RNFL thinning at the 10 o'clock position in the right eye and at the 2 o'clock position in the left eye.

**Figure 4:**
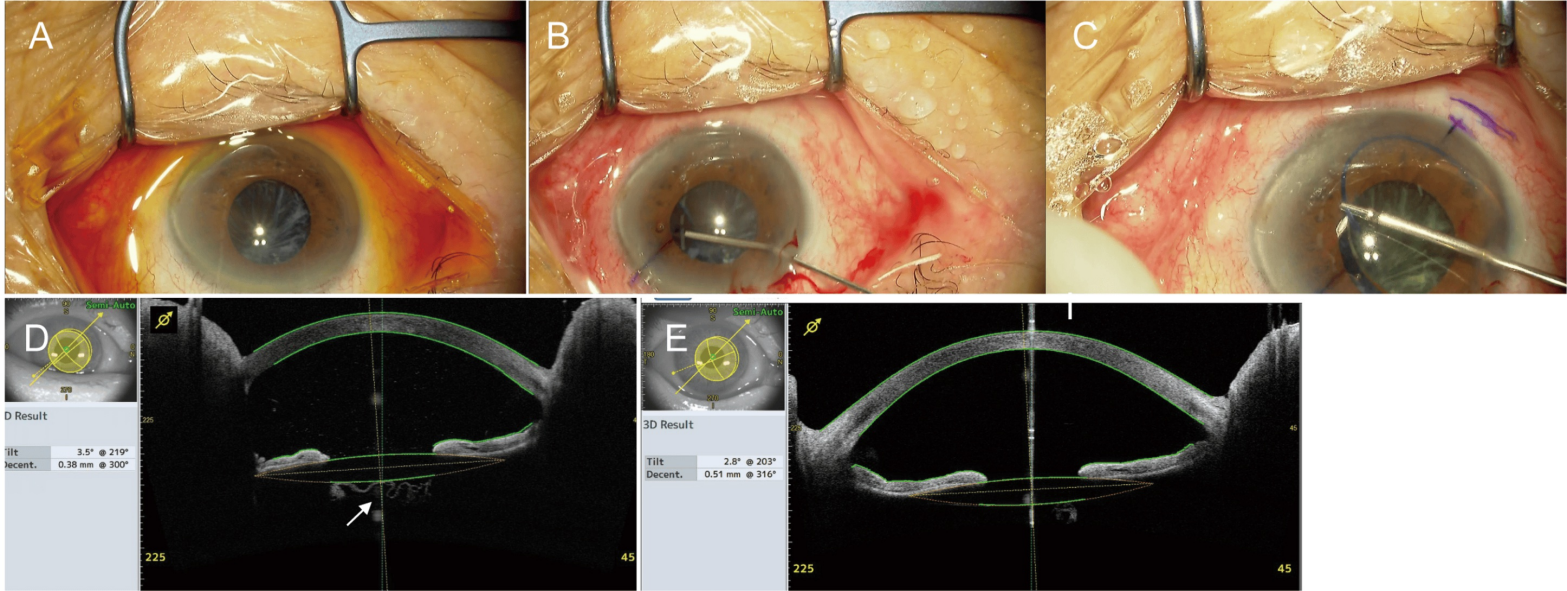
Intraoperative observations during intrascleral intraocular lens (IOL) fixation surgery in Case 3 and postoperative findings after intrascleral IOL fixation surgery in Case 3. (A–C) IOL intrascleral fixation was performed seven weeks after the initial cataract surgery (phacoemulsification). (A) Findings at the beginning of surgery. The capsule remained in place, with some shrinkage and opacity. (B) The space between the capsule and the iris was created using viscoelastic material. (C) After a three-piece IOL was inserted using an injector, a 30-G needle was inserted 2 mm from the limbus such that the needle would exit between the lens capsule and the iris, and the IOL was fixed intrasclerally using the double-needle technique. (D,E) Postoperative findings after intrascleral IOL fixation surgery in Case 3. Anterior segment optical coherence tomography (OCT) image obtained on postoperative day 11 (D) shows capsular opacity over the visual axis (arrow). Anterior segment OCT image (E) obtained at six months postoperatively. No capsular opacity was observed over the visual axis. The anterior segment OCT image reveals an IOL tilt (Tilt) of 2.8° and decentralization (Decent.) of 0.51 mm without iris capture by the IOL (E).

Seven weeks later, the patient underwent intrascleral fixation of the IOL using the double-needle technique at our hospital, in which a 30-G needle was carefully inserted and maneuvered such that it would exit the eye between the capsule and the iris (Figure 4) (Video 3). Intraoperatively, no vitreous body prolapse was observed; therefore, vitrectomy was not performed, and the lens capsule was left intact. On the day following the second surgery, the IOP was 22 mmHg, prompting the initiation of 0.5% timolol maleate eye drops. Five days after the second surgery, the corneal endothelial cell density was 1403 cells/mm^2^, and the BCVA in the right eye was 20/2000, with an IOP of 11 mmHg under 0.5% timolol maleate eye drops. On postoperative day 11, the BCVA of the operated eye was 20/600 owing to capsular opacity over the visual axis (Figure 4D, arrow). At both 19 days and 10 weeks postoperatively, YAG incisions were made over the visual axis to address the opacity of the capsule. Postoperatively, good IOL fixation was achieved without inadvertent iris capture by the IOL. Six months after the surgery, anterior segment OCT revealed an IOL tilt of 2.8° and decentralization of 0.51 mm (Figure 4E). Good visual acuity was observed after YAG irradiation. The BCVA of the operated eye at six months postoperatively was 20/20. Six months after the surgery, the corneal endothelial cell density was 1347 cells/mm^2^, representing an overall decrease of 31.6% compared to the preoperative value.

**Video 3:** Intrascleral intraocular lens fixation with lens capsule preservation for aphakic eyes in Case 3.

No serious complications, such as retinal detachment or vitreous hemorrhage, were observed in any of the three patients in this study.

## Discussion

This report describes the cases of three older adult patients diagnosed with PEX who underwent initial cataract surgery without IOL implantation. The surgical procedure was completed with the eyes rendered aphakic, as the weakened zonular fibers associated with PEX precluded secure intracapsular fixation of the IOL. Subsequently, secondary intrascleral fixation of the IOL was performed immediately anterior to the capsule. The double-needle technique was used in all three patients without excision of the capsule and in two patients without excision of the vitreous body. No severe complications, such as retinal detachment and vitreous hemorrhage, were observed in any of the three patients in this study.

Previous reports [6,7] have described a combined approach involving PEA and intrascleral IOL fixation with capsule preservation. This technique typically involves inserting a CTR followed by capturing the IOL optics using the anterior capsulorhexis opening. However, this method was not applicable to the patients in this case series. The reason for this lies in the nature of a two-stage procedure, such as that used herein, where it is often difficult to capture the optics because the anterior capsulorhexis opening tends to contract when performed six to eight weeks after the initial surgery. In addition, in this case series, CTR insertion proved challenging because of the weakness and dehiscence of the zonules.

Despite these challenges, even in situations akin to those in the present cases where CTR insertion and IOL optics capture with the anterior capsulorhexis opening are impossible, there are several potential advantages of the capsule-preserving method. These advantages include the following: (1) reduced risk of intraoperative IOL drop; (2) increased possibility of vitreous preservation, thereby avoiding unnecessary vitrectomy and capsule resection and ensuring a less-invasive surgery; (3) shortened operative time; and (4) capsule preservation, which may prevent fluttering of the iris and associated IOL capture.

The flow of aqueous and vitreous humors through the gap around the optics of the IOL during eye movement causes the iris to flutter back and forth. In eyes lacking a capsule, iris fluttering is frequently observed [5]. Iris fluttering leads to iris capture of the IOL, one of the major complications of intrascleral IOL fixation [5,8]. The occurrence of iris capture with intrasclerally fixated IOLs has been reported in 8%-8.3% [3,9] of cases, and peri-iridotomy is recommended to avoid the risk of iris capture [3]. Preservation of the capsule is considered to inhibit iris fluttering, thus preventing iris capture of the IOL.

One concern with the capsule-preserving approach is that it is difficult to fix the IOL immediately in front of the capsule. During surgery, it is possible that the IOL cannot be centrally fixed or may tilt due to obstruction by the capsule. However, in the three patients in this report, adequate fixation of the IOL immediately in front of the capsule could be performed. The tilts of the IOL were 2.2°, 8.1°, and 2.8° in patients 1, 2, and 3, respectively, which were comparable to those reported previously (3.8°-8.8°) [3,10-15], although it is worth noting that an IOL tilt exceeding 10° is considered to cause refractive error [10]. The IOL eccentricities in patients 1, 2, and 3 were 0.50, 0.56, and 0.51 mm, respectively, which were comparable to the IOL eccentricity in prior reports (0.39-0.60 mm) [10,12-16]. It has been reported that a decentration greater than 0.4 mm [17,18] and a tilt of 7° or more [17,18], or 10° or more [10], can adversely affect visual function. The IOL tilt and the decentration observed with this surgical technique were within acceptable limits and were not considered to impact visual function.

Moreover, if the surgeon lacks experience in managing complex cataract cases, and there is a high likelihood that successful IOL implantation may not be achieved due to underestimating the preoperative findings of PEX, it would be prudent to postpone the surgery for a few weeks and opt for scleral fixation later, rather than proceeding with undue haste. Even for experienced cataract surgeons, same-day intrascleral IOL fixation may not be feasible due to scheduling constraints or limitations in surgical facilities and equipment. In such cases, deferring the intrascleral IOL fixation for a few weeks can be beneficial.

As previously discussed, the primary advantage of scleral IOL fixation without capsule preservation is the removal of both the vitreous and lens capsule during the procedure, which eliminates physiological opacities and generally results in improved visual acuity. In contrast, a significant disadvantage of the capsule-preserving method is the slower visual recovery. The preserved lens capsule in this method is prone to opacification due to the dual surgical interventions and ensuing postoperative inflammation, leading to diminished postoperative vision and necessitating a YAG laser posterior capsulotomy.

According to a previous study, the incidence of posterior capsular opacification (PCO) following single-stage IOL scleral fixation with lens capsule preservation was 5.4% (two eyes), both of which required posterior capsulotomy [7]. However, there are no reported cases in the literature of IOL scleral fixation for aphakic eyes with capsule preservation, as performed in this study, where the procedure was conducted in two stages. In all cases of two-stage IOL intrascleral fixation with capsule preservation, 100% of patients experienced lens capsule opacification, necessitating a YAG capsulotomy. The significantly higher rate of capsule opacification compared to previous reports of single-stage surgeries is likely attributable to the increased likelihood of capsular opacification following two surgical interventions and the associated postoperative inflammation.

Additionally, this approach was associated with prolonged postoperative inflammation and elevated IOP, possibly due to the presence of residual capsular and lens epithelial cells, as well as PEX, as observed in Case 3.

A decrease in corneal endothelial density of 6.5%, 47.9%, and 31.6% was observed in Cases 1, 2, and 3, respectively, following the second surgery compared to the pre-second surgery measurements taken after the initial procedure. This decrease tended to be higher in Cases 2 and 3 compared with that in previously reported cases (6% [19] and 12.5% [20] at three months). Possible explanations for this difference include surgical invasion due to additional anterior vitrectomy (Case 2) and prolonged postoperative inflammation and elevated IOP due to PEX (Case 3). The rate of corneal endothelial loss in Case 1 was 6.5%, which was consistent with that reported previously [19,20].

It is possible that the preserved lens capsule may drop over a long-term postoperative period. Although we did not observe dislocation or dropping of the preserved lens capsule in our patients' six-month to one-year postoperative follow-up period, it is necessary to continue monitoring for lens capsule drop in the long term.

## Conclusions

In summary, the results from our case series indicate that for aphakic eyes in which the initial cataract surgery is terminated due to difficulty in IOL fixation within the capsule, arising from zonule weakness, a second surgery involving intrascleral IOL fixation with capsule preservation can be advantageous. This remains true even if the optics of the IOL cannot be captured through the anterior CCC of the preserved capsule due to contraction of the anterior CCC. This approach has the potential to prevent intraoperative vitreous prolapse and minimize surgical invasiveness. Moreover, retaining the remaining capsule may prevent iris fluttering as well as subsequent pupillary IOL capture. Furthermore, secondary IOL scleral fixation offers a viable option in situations where immediate IOL scleral fixation cannot be performed due to scheduling constraints, limitations in surgical equipment, or for surgeons who are less experienced with complex cases. This approach allows the procedure to be safely postponed for several weeks, providing time for adequate preparation and ensuring optimal surgical outcomes. Hence, preserving the capsule in secondary IOL intrascleral fixation surgery for aphakic eyes is a meaningful and feasible approach.
